# α-Synuclein triggers cofilin pathology and dendritic spine impairment via a PrP^C^-CCR5 dependent pathway

**DOI:** 10.1038/s41419-024-06630-9

**Published:** 2024-04-13

**Authors:** Marina I. Oliveira da Silva, Miguel Santejo, Isaac W. Babcock, Ana Magalhães, Laurie S. Minamide, Seok-Joon Won, Erika Castillo, Ellen Gerhardt, Christiane Fahlbusch, Raymond A. Swanson, Tiago F. Outeiro, Ricardo Taipa, Michael Ruff, James R. Bamburg, Márcia A. Liz

**Affiliations:** 1 Neurodegeneration Team, Nerve Regeneration Group, IBMC -Instituto de Biologia Molecular e Celular and i3S - Instituto de Investigação e Inovação em Saúde, University of Porto, 4200-135 Porto, Portugal; 2 Department of Biochemistry and Molecular Biology, Colorado State University, Fort Collins, CO 80523 USA; 3 Addiction Biology Group, IBMC -Instituto de Biologia Molecular e Celular and i3S - Instituto de Investigação e Inovação em Saúde, University of Porto, 4200-135 Porto, Portugal; 4 Department of Neurology, University of California, San Francisco, CA 94158 USA; 5 Department of Experimental Neurodegeneration, Center for Biostructural Imaging of Neurodegeneration, University Medical Center Göttingen, 37073 Göttingen, Germany; 6 Max Planck Institute for Multidisciplinary Sciences, 37077 Göttingen, Germany; 7 Translational and Clinical Research Institute, Faculty of Medical Sciences, Newcastle University, Framlington Place, Newcastle Upon Tyne, NE2 4HH UK; 8 Scientific employee with an honorary contract at Deutsches Zentrum für Neurodegenerative Erkrankungen (DZNE), 37075 Göttingen, Germany; 9 Neuropathology Unit, Centro Hospitalar Universitário de Santo António, 4099-001 Porto, Portugal; 10 Autoimmune and Neuroscience Research Group, UMIB – Unit for Multidisciplinary Research in Biomedicine, ICBAS – School of Medicine and Biomedical Sciences, University of Porto, 4050-313 Porto, Portugal; 11 ITR – Laboratory for Integrative and Translational Research in Population Health, 4050-600 Porto, Portugal; 12 Creative Bio-Peptides, Rockville, MD 20854 USA

**Keywords:** Cell biology, Cell death

## Abstract

Cognitive dysfunction and dementia are critical symptoms of Lewy Body dementias (LBD). Specifically, alpha-synuclein (αSyn) accumulation in the hippocampus leading to synaptic dysfunction is linked to cognitive deficits in LBD. Here, we investigated the pathological impact of αSyn on hippocampal neurons. We report that either αSyn overexpression or αSyn pre-formed fibrils (PFFs) treatment triggers the formation of cofilin-actin rods, synapse disruptors, in cultured hippocampal neurons and in the hippocampus of synucleinopathy mouse models and of LBD patients. In vivo, cofilin pathology is present concomitantly with synaptic impairment and cognitive dysfunction. Rods generation prompted by αSyn involves the co-action of the cellular prion protein (PrP^C^) and the chemokine receptor 5 (CCR5). Importantly, we show that CCR5 inhibition, with a clinically relevant peptide antagonist, reverts dendritic spine impairment promoted by αSyn. Collectively, we detail the cellular and molecular mechanism through which αSyn disrupts hippocampal synaptic structure and we identify CCR5 as a novel therapeutic target to prevent synaptic impairment and cognitive dysfunction in LBD.

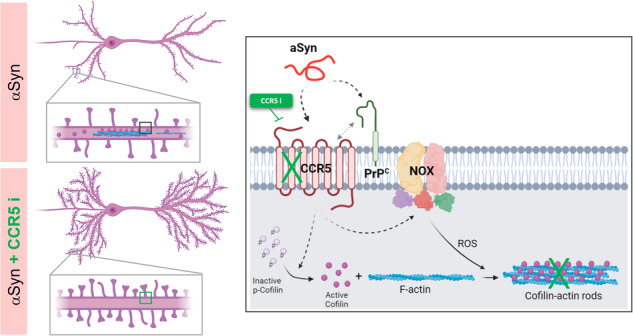

## Introduction

α-Synuclein (αSyn), well-known for its involvement in Parkinson’s disease (PD) [1–3], is implicated in other synucleinopathies, namely Lewy Body dementias (LBD), which include Dementia with Lewy Bodies (DLB) and PD with dementia (PDD). DLB accounts for approximately 30% of all age-related dementias [4]. In PD, 20–40% of the patients have cognitive impairments at disease onset, and ∼80% of the patients develop PDD with the course of the disease, which massively affects the quality of life. These two disorders present similar cognitive symptomatology implying that disease-modifying therapies will be effective in both diseases. Until now, no treatments have been proven to slow or stop disease progression in LBD, indicating that new approaches to target pathophysiological mechanisms are needed. Synapse destruction, occurring significantly before neuronal loss, underlies cognitive deficits and dementia in LBD [5], indicating that treatments that protect and restore synapses might be a central approach for halting LBD [6].

Multiplications of the *SNCA* gene have been described in LBD cases, whose severity of cognitive impairment and age of onset correlates with *SNCA* copy number [7]. The hippocampus, a brain region that plays key roles in memory and learning, is one of the most affected regions by αSyn pathology [8]. In this respect, hippocampal volume loss is observed in DLB and PDD patients, but not in cases of PD with normal cognition [9]. Moreover, increased levels of αSyn pathology are observed in post-mortem hippocampal tissue of LBD cases [10]. These observations suggest that the impact of αSyn on the hippocampus may underlie cognitive deficits observed in LBD. Additionally, the reported synaptic dysfunction in the hippocampus of mouse models of synucleinopathies strengthens the connection between cognitive deficits and hippocampal LB pathology.

In this study, we aimed to characterize the cellular and molecular mechanism underlying hippocampal pathology downstream of αSyn. We focused on the actin cytoskeleton, not only due to its critical role in synaptic function but also because a link between actin dysregulation and αSyn has been increasingly supported by the literature [11–13]. One of the consequences of the dysregulation of the actin cytoskeleton in neurodegenerative disorders is the formation of cofilin-actin rods [14–16]. These are structures composed of bundles of cofilin-saturated actin filaments, which result from localized cofilin hyperactivation by dephosphorylation and oxidation [17]. Cofilin-actin rods have been mainly implicated in cognitive impairment in Alzheimer’s disease (AD) [18–20], and were shown to block intracellular trafficking and induce synaptic loss in cultured hippocampal neurons [21]. In AD, the formation of these structures was suggested to be mediated by pathways involving cellular prion protein (PrP^C^)/NADPH oxidase (NOX) and the chemokine receptors CXCR4 and CCR5 [22, 23]. Importantly, CCR5 was shown to have a negative impact on neuronal plasticity, which is crucial for memory and learning [24, 25].

Here, we show that αSyn induces cofilin pathology in hippocampal neurons via a cellular PrP^C^-CCR5 dependent pathway. Moreover, cofilin dysregulation mediates dendritic spine impairment in response to αSyn, which is rescued by CCR5 inhibition. Based on the present data, we propose a novel action of CCR5 to exert allosteric regulation of the αSyn-activated neuronal PrP^C^/NOX complex that elicits a pathological cofilin rod response resulting in spine disruption. Antagonists of CCR5 may therefore protect synapses to provide treatment for cognitive impairment in LBD.

## Results

### αSyn overexpression induces cofilin-actin rod formation in hippocampal neurons

**Fig. 1:**
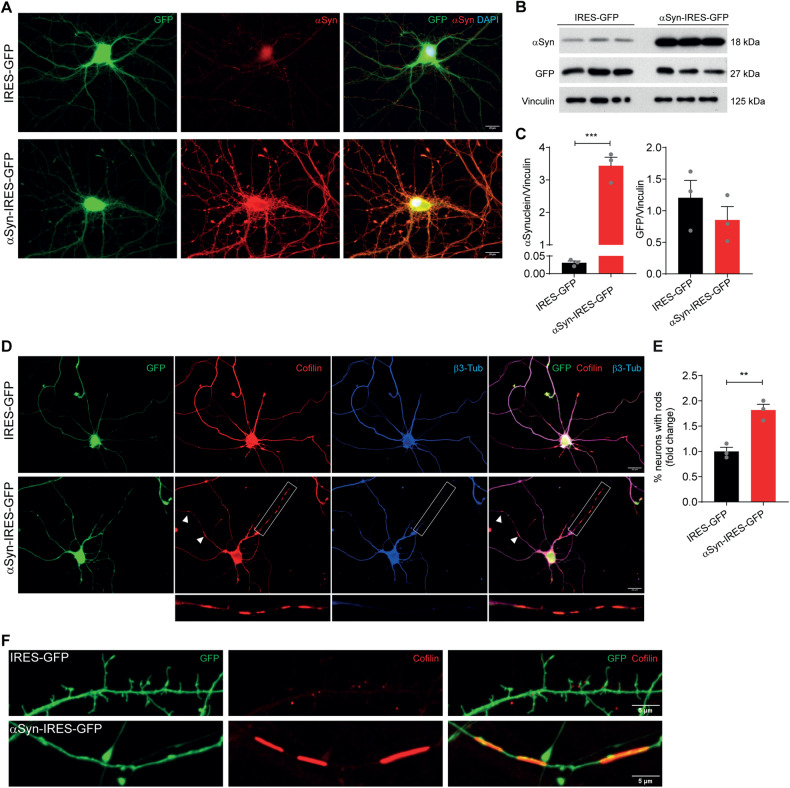
αSyn overexpression induces cofilin-actin rod formation in hippocampal neurons. **A** Representative images of DIV14 hippocampal neurons expressing GFP or αSyn and immunostained with αSyn (red). Scale bar: 20 μm. **B**, **C** Representative western blot (**B**) and respective quantification (**C**) of α-Syn and GFP levels in DIV14 hippocampal neurons expressing GFP or αSyn. Vinculin was used as loading control. Data represent mean±SEM (*n* = 3 independent samples/condition). ***p < 0.001 by Student’s *t* test. **D** Representative images of GFP- or αSyn-expressing hippocampal neurons immunostained for cofilin (red) and β3-tubulin (blue). Scale bar: 20 μm. Insets and arrowheads indicate cofilin-actin rod structures in αSyn-expressing neurons. **E** Quantification of the percentage of neurons with rods (shown as fold change relative to control) relative to **D**. Data represent mean±SEM (*n* = 3 independent experiments with ≥100 neurons/condition/experiment). **p < 0.01 by Student’s *t* test. **F** Representative images of dendrites of GFP- or αSyn-expressing hippocampal neurons immunostained for cofilin (red). Scale bar: 5 μm.

To characterize downstream mechanisms underlying hippocampal dysfunction in LBD, we analyzed the effect of overexpressing wild-type (WT) αSyn in primary cultures of hippocampal neurons. This way, we aimed to establish a scenario of increased levels and aggregation of αSyn [26], which were shown to induce synaptic dysfunction [27]. To drive αSyn overexpression we infected rat hippocampal neurons with lentivirus encoding for WT αSyn-IRES-GFP or IRES-GFP as control. αSyn overexpression was initially confirmed in DIV7 αSyn-expressing neurons by western blot and immunocytochemistry (Supplementary Fig. S1A, B). In αSyn-expressing neurons the protein was highly phosphorylated at Ser129, mimicking the aberrant accumulation of αSyn pS129, a marker for αSyn aggregation in the brain of patients with LBD [28] (Supplementary Fig. S1A, B). At DIV14, a time point when endogenous αSyn is already expressed and enriched in presynaptic terminals of hippocampal neurons, we confirmed αSyn overexpression (Fig. 1A) and determined that its levels were increased approximately 3-fold when compared to control cells expressing similar levels of GFP (Fig. 1B, C). These results confirm that we have successfully established a cell system of αSyn hippocampal pathology.

Cofilin-actin rods are one of the features associated with hippocampal pathology in response to Aβ, leading to synaptic impairment and cognitive dysfunction in AD [29]. We hypothesized that αSyn would exert a similar effect in the context of hippocampal pathology. Using our established cell system of αSyn hippocampal pathology, we demonstrated that αSyn overexpression induced a 1.8-fold increase in the percentage of neurons presenting rods when compared to control neurons (Fig. 1D, E). Interestingly, the percentage of neurons with rods in WT αSyn-transduced neurons was approximately of 20%, a similar value to the one reported for Aβ- and TNFα-induced rods in rat hippocampal neurons [30, 31]. Strikingly, rod-containing neurites in αSyn-expressing neurons were devoid of βIII-tubulin staining, suggesting a disturbance of the cytoskeleton integrity (Fig. 1D, insets), and of dendritic spines (Fig. 1F). These results show that αSyn-induced cofilin pathology mediates dendritic spine impairment in hippocampal neurons.

### Cofilin hippocampal pathology manifests in cognitively impaired αSyn transgenic mice and LBD patients

**Fig. 2:**
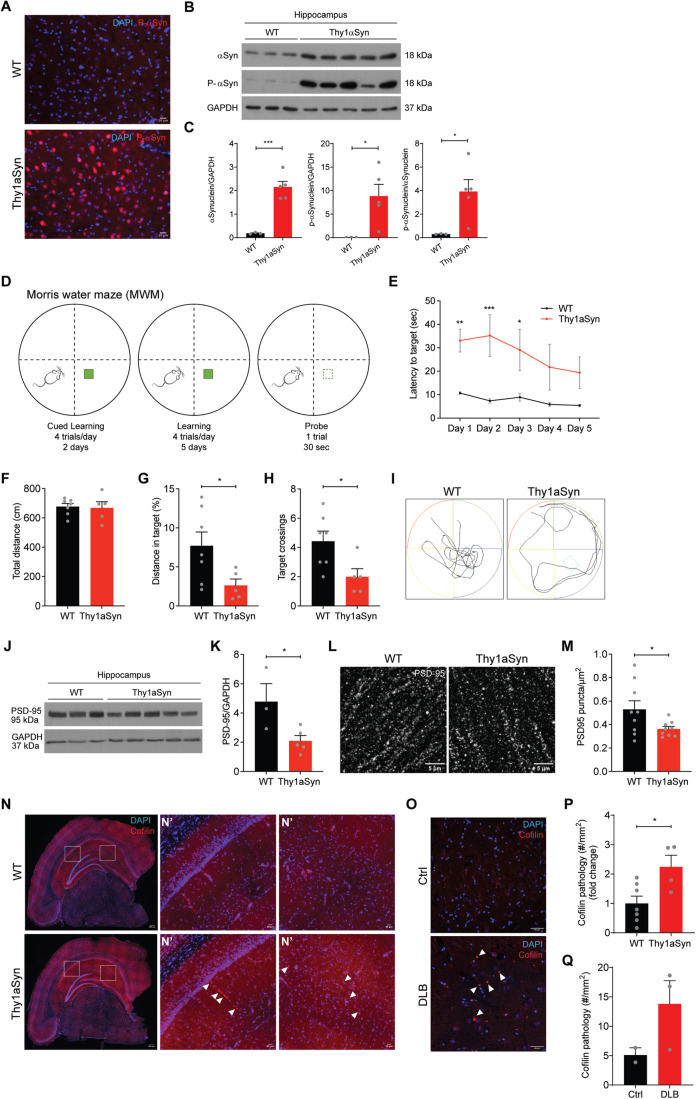
Hippocampal cofilin pathology is recapitulated in Thy1-aSyn mice with cognitive impairment and in DLB patients. **A** Representative images of brain sections from 6-month-old WT and Thy1-aSyn mice immunostained for αSyn pS129 (red). DAPI (blue). Scale bar: 20 μm. **B**, **C** Western blot analysis (**B**) and respective quantification (**C**) of αSyn and αSyn pS129 levels in hippocampus from WT and Thy1-aSyn mice. GAPDH was used as loading control. Data represent mean±SEM (*n* = 3–5 animals/genotype). *p < 0.05, ***p < 0.001 by Student’s *t* test. **D**–**I** Morris Wa*t*er Maze (MWM) test in 6-month-old animals. **D** Schematic representation of the MWM test. **E** Latency to target in the learning phase. Data represent mean±SEM (*n* = 5–7 animals/genotype). *p < 0.05, **p < 0.01, ***p < 0.001 by Student’s *t* test. **F**–**I** Probe trial with analyses of the total distance (**F**), distance in target (**G**), target crossings (**H**), and a representative track of the WT and Thy1-aSyn mice during the probe test (**I**). Data represent mean±SEM (*n* = 5–7 animals/genotype). *p < 0.05 by Student’s *t* test. **J**, **K** Western blot analysis (**J**) and respective quantification (**K**) of PSD-95 levels in the hippocampus of 6-month-old WT and Thy1-aSyn mice. GAPDH was used as loading control. Data represent mean±SEM (*n* = 3–5 animals/genotype). *p < 0.05 by Student’s *t* test. **L** Representative images of the hippocampal region of brain sections from 6-month-old WT and Thy1-aSyn mice immunostained for PSD-95 (white). Scale bar: 5 μm. **M** PSD-95 puncta analysis per μm^2^ in the hippocampus region relative to **L**. Data represent mean±SEM (*n* = 9 animals/genotype). *p < 0.05 by Student’s *t* test. **N** Representative images of brain sections from 6-month-old WT and Thy1-aSyn mice immunostained for cofilin (red). DAPI (blue). Scale bar: 200 μm. **N**’ Zoom-ins from **N**. Scale bar: 20 μm. Arrowheads indicate cofilin-actin rod structures. **O** Representative images of brain sections from Control and DLB patients immunostained for cofilin (red). DAPI (blue). Scale bar: 50 μm. **P** Hippocampal cofilin pathology evaluated by the number of cofilin aggregates and rods per mm^2^ (fold change relative to WT) relative to **N**. Data represent mean±SEM (*n* = 4–7 animals/genotype). *p < 0.05 by Student’s *t* test. **Q** Hippocampal cofilin pathology evaluated by the number of cofilin aggregates and rods per mm^2^ relative to **O**. Data represent mean±SEM (*n* = 2–3 patients/condition).

Following our in vitro findings, we aimed to verify if αSyn-induced hippocampal cofilin pathology was recapitulated in vivo. We used a mouse model overexpressing human WT αSyn under the control of the neuronal Thy-1 promoter (Thy1-aSyn mice) [32]. This model recapitulates the αSyn levels observed in patients with multiplications of the *SNCA* gene and presents synaptic and memory impairments starting at early stages, similar to what is seen in PD patients who develop dementia. Using 6-month-old animals, an age when cognitive dysfunction in Thy1-aSyn was reported [33], we initially confirmed the overexpression of human αSyn in the hippocampus as well as the presence of its pathologic-associated form αSyn pS129, by immunostaining and western blot (Fig. 2A–C).

We followed by validating cognitive impairment in the Thy1-aSyn mice. We evaluated hippocampal-related cognitive functions namely spatial memory assessed by the Morris Water Maze (MWM) test. In the MWM test (Fig. 2D–I), 6-month-old Thy1-aSyn mice presented defects in the learning phase showing increased latency to find the platform (Fig. 2E). In the probe test, although Thy1-aSyn mice traveled the same distance as WT littermates (Fig. 2F), they showed a decreased distance in the target square (Fig. 2G, I) and made fewer target crossings (Fig. 2H, I). Additionally, we have also evaluated hippocampal-related cognitive functions regarding non-spatial memory tested by Normal Object Recognition (NOR). In NOR test (Supplementary Fig. S2A–C), the total time of exploration of the objects was not different between the two experimental groups (Supplementary Fig. S2B). However, whereas WT mice spent more time exploring the new object, Thy1-aSyn mice did not distinguish between the familiar and new objects, as measured by the discrimination index (Supplementary Fig. S2C). These results validate the decreased recognition memory which was previously reported for the Thy1-aSyn mice [32]. We also confirmed a decreased body weight gain in the Thy1-aSyn mice (Supplementary Fig. S2D), as indicated in previous studies [32].

Following behavioral tests, we investigated whether impaired hippocampal-related memory was accompanied by synaptic impairment in Thy1-aSyn mice, by measuring the levels of the post-synaptic protein PSD-95 in the hippocampus. Our results showed decreased PSD-95 levels in Thy1-aSyn mice when compared with WT controls, both by western blot (Fig. 2J, K) and by immunohistochemistry (Fig. 2L, M).

Having a model of hippocampal αSyn pathology provided an excellent tool to confirm in vivo whether αSyn-induced cofilin pathology plays a role in hippocampal synaptic dysfunction and cognitive deficits in LBD. Supporting our hypothesis, we validated the presence of cofilin-actin rods in the hippocampus of 6-month-old Thy1-aSyn mice, with a 2.2-fold increase in cofilin pathology (cofilin-actin rods and aggregates) compared with WT littermates (Fig. 2N, P). In the *substantia nigra*, a region majorly affected in synucleinopathies, no cofilin pathology was observed (Supplementary Fig. S2E), although robust levels of αSyn are expressed in that brain region in the Thy1-aSyn mice [32, 34]. Interestingly, similarly to what we have observed in the synucleinopathy mouse model, cofilin immunostaining of DLB patient brain slices evidenced the presence of cofilin-actin rod structures in the hippocampal region (Fig. 2O, Q). Taken together, our in vivo data indicates that hippocampal cofilin pathology is associated with synaptic defects and cognitive dysfunction in LBD.

### Cofilin-actin rod formation is recapitulated in a model of αSyn PFF-induced pathology

**Fig. 3:**
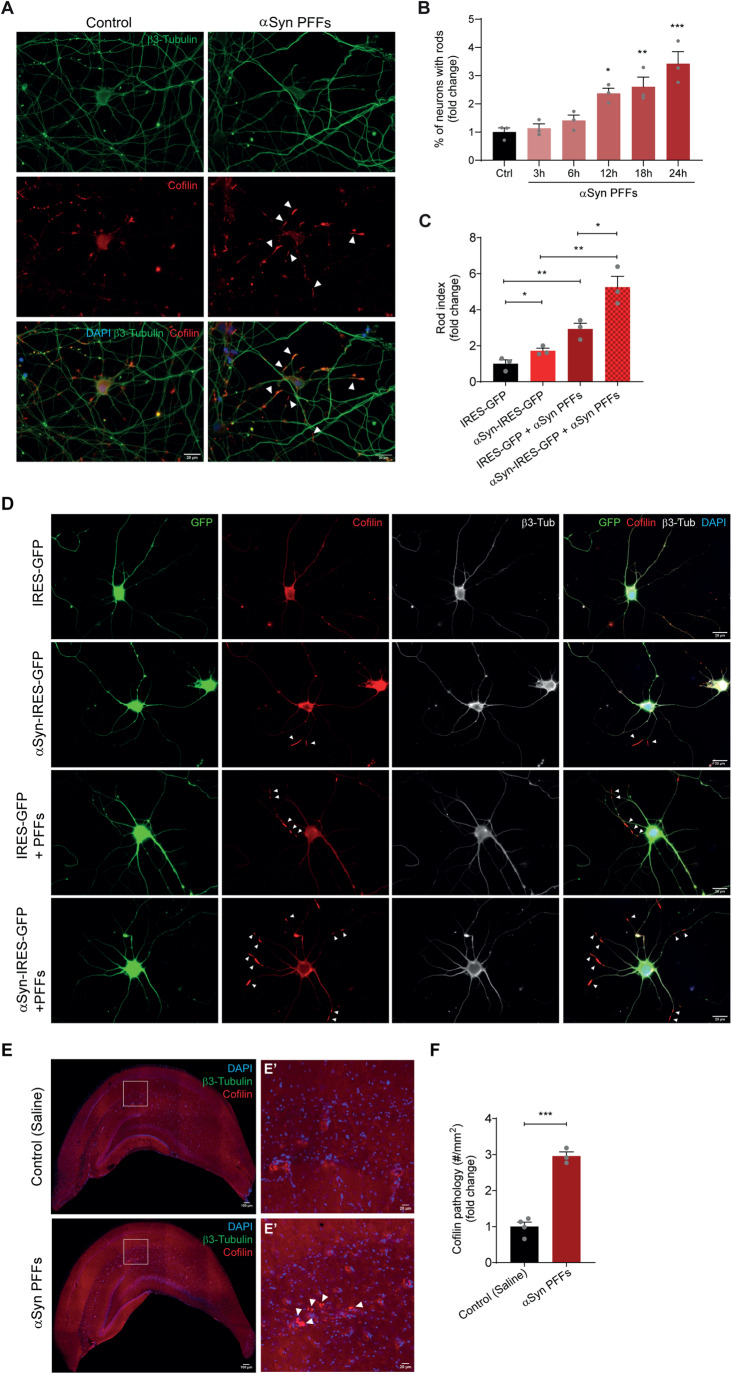
αSyn pre-formed fibrils induce cofilin-actin rods in hippocampal neurons. **A** Representative images of DIV7 hippocampal neurons pre-treated with control (PBS) or αSyn PFFs for 24 h and immunostained for β3-tubulin (green) and cofilin (red). Scale bar: 20 μm. Arrowheads indicate rod structures. **B** Percentage of neurons with rods (fold change relative to control) of DIV7 hippocampal neurons pre-treated with control (PBS) or αSyn PFFs for 3 h, 6 h, 12 h, 18 h, and 24 h. Data represent mean±SEM (*n* = 3 independent experiments with ≥100 neurons/condition/experiment). *p < 0.05, **p < 0.01, ***p < 0.001 by One-way ANOVA with Dunnett’s multiple comparisons test. **C** Rod index (fold change relative to control) relative to **D**. Data represent mean±SEM (*n* = 3 independent samples/condition with ≥100 neurons/sample). *p < 0.05, **p < 0.01 by Student’s *t* test. **D** Representative images of DIV14 hippocampal neurons expressing GFP or αSyn, untreated or pre-treated at DIV7 with 150 ng/mL of αSyn PFFs, and immunostained at DIV14 for β3-tubulin (white) and cofilin (red). Scale bar: 20 μm. Arrowheads indicate rod structures. **E** Representative images of hippocampal brain sections from control (Saline) and αSyn PFFs injected WT mice 6 months post-injection immunostained for cofilin (red). DAPI (blue). Scale bar: 200 μm. **E**’ Zoom-ins from E. Scale bar: 20 μm. Arrowheads indicate cofilin-actin rod structures. **F** Cofilin pathology evaluated by the number of cofilin aggregates and rods per mm^2^ (fold change relative to WT) in the hippocampal region relative to **E**. Data represent mean±SEM (*n* = 3–4 animals/condition). ***p < 0.001 by Student’s *t* test.

To further investigate the effect of αSyn on hippocampal neurons we tested a model of exogenous addition of αSyn preformed fibrils (PFFs). αSyn PFFs, which derive from the aggregation of recombinant αSyn monomers, were shown to be able to propagate the misfolding and aggregation of αSyn in a prion-like manner in the recipient cells promoting changes in neurons and mice similar to those observed in PD [35, 36]. We followed the progression of cofilin-actin rod formation upon addition of αSyn PFFs to DIV7 hippocampal neurons over 24 h. Cofilin-actin rods did not increase significantly at 3 h or 6 h after the addition of αSyn PFFs. However, by 12 h of exposure to αSyn PFFs there was a 2.3-fold increase in the percentage of neurons with rods which continued to increase at 18 h (2.6-fold) and 24 h (3.4-fold; Fig. 3A, B). These data suggest an extracellular effect of αSyn PFFs on rod induction, which seems unrelated to seeding and intracellular αSyn aggregation, as DIV7 hippocampal neurons do not show detectable levels of endogenous αSyn (Supplementary Fig. S1A, B).

To analyze the generation of rods when endogenous αSyn is detectable, we analyzed the effect of αSyn PFFs on DIV14 neurons. Moreover, to potentiate the previously reported seeding process [37], we combined αSyn overexpression with αSyn PFF addition. Hippocampal neurons expressing αSyn were pre-treated with 150 ng/mL of αSyn PFFs at DIV7 and analyzed for rods at DIV14. As expected, αSyn overexpression induced cofilin-actin rods in mature neurons as observed by the 1.7-fold increase in rod index (Fig. 3C, D). αSyn PFFs induced a 2.9-fold increase in rod index in control GFP-expressing cells while in αSyn-expressing neurons the increment in rods was approximately 3-fold (when compared to αSyn-transduced neurons; Fig. 3C, D). In this experiment, we detected by immunocytochemistry high levels of αSyn pS129 in αSyn-expressing neurons and GFP-expressing neurons treated with αSyn PFFs, but the highest levels of αSyn pS129 were observed in αSyn-expressing neurons also treated with αSyn PFFs (Supplementary Fig. S3A). Collectively, these experiments suggest that the intracellular accumulation of αSyn pS129 enhances the formation of rod structures. However, we cannot rule out the possibility of alternative explanations such as effects from extracellular αSyn PFFs or their uptake.

To assess whether αSyn PFFs have an effect on cofilin-actin rods in vivo, we used a model of αSyn PFF injection into the striatum of WT mice. This model was chosen to analyze the effect of spreading in vivo, as αSyn species injected in the striatum trigger endogenous αSyn aggregation in the cortex, thalamus, and hippocampus [36–38]. Confirming the model, we observed the presence of αSyn pS129 in the hippocampus of WT mice 6 months post-αSyn PFF injection (Supplementary Fig. S3B). Importantly, αSyn PFF injected-mice presented a 2.9-fold increase in cofilin pathology in the hippocampus when compared to vehicle-injected mice (Fig. 3E, F). These results recapitulate the in vitro data showing αSyn PFF-induced rods in hippocampal neurons. More importantly, these findings further support that the spreading of αSyn species also contributes to cofilin pathology.

### PrP^C^ and CCR5 are linked in the molecular mechanism of αSyn-induced cofilin pathology

**Fig. 4:**
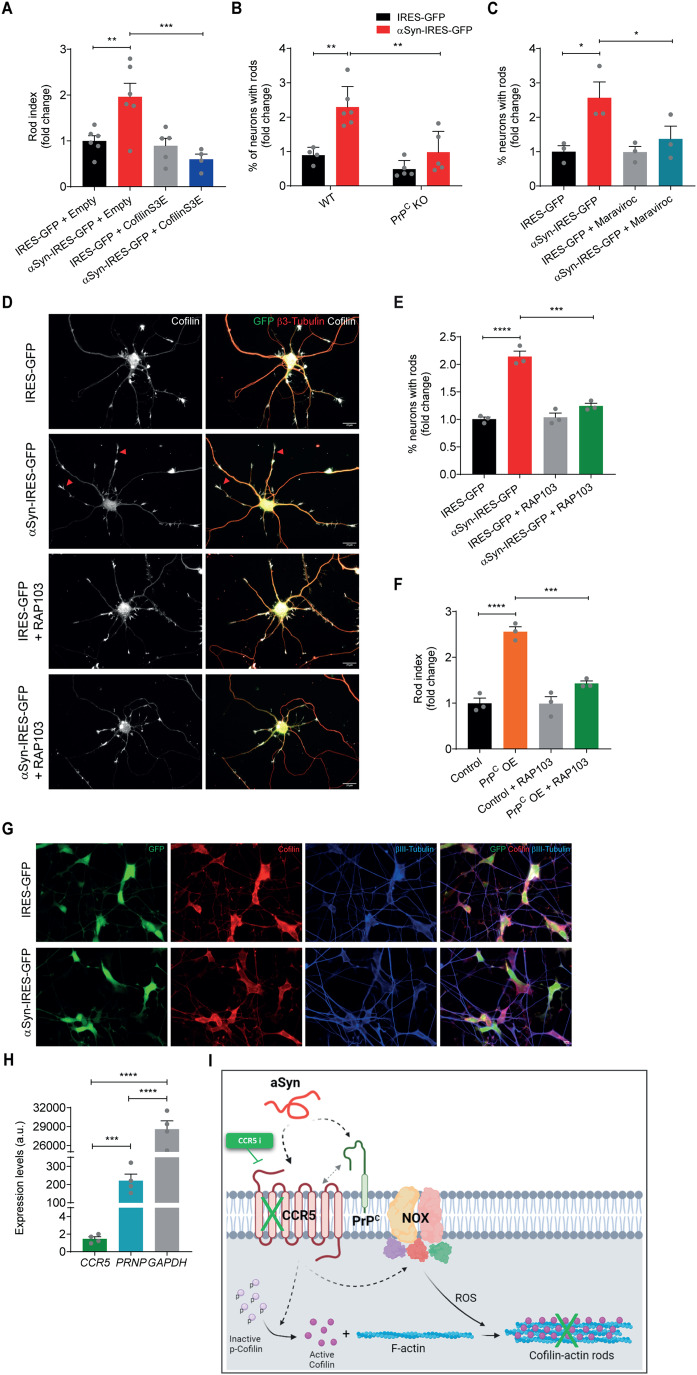
A PrP^C^-CCR5 pathway mediates αSyn-induced rod formation*.* **A** Rod index quantification (fold change relative to control) of DIV14 hippocampal neurons expressing GFP or αSyn either with pmRFP-N1 (empty) or cofilin-S3E. Data represent mean±SEM (*n* = 4–6 independent samples/condition with an average of 25 neurons/sample). **p < 0.01, ***p < 0.001 by One-way ANOVA with Sidak’s multiple comparisons test. **B** Quantification of the percentage of neurons with rods (fold change relative to control) in DIV7 hippocampal neurons from WT or PrP^C^ KO mice expressing GFP or αSyn. Data represent mean±SEM (*n* = 4–6 independent samples/condition with ≥100 neurons/sample). **p < 0.01 by Two-way ANOVA with Sidak’s multiple comparison test. **C** Quantification of the percentage of neurons with rods in DIV7 hippocampal neurons expressing GFP or αSyn and pre-treated for 24 h with 50 nM Maraviroc. Data represent mean±SEM (*n* = 3 independent experiments with ≥100 neurons/condition/experiment). *p < 0.05 by One-way ANOVA with Sidak’s multiple comparisons test. **D** Representative images of DIV7 hippocampal neurons expressing GFP or αSyn and pre-treated for 24 h with 50 pM RAP-103. Immunostaining for β3-tubulin (red) and cofilin (white). Scale bar: 20 μm. Arrowheads indicate rod structures. **E** Quantification of the percentage of neurons with rods (shown as fold change relative to control) relative to **D**. Data represent mean ± SEM. (*n* = 3 independent experiments with ≥100 neurons/condition/experiment). ***p < 0.001, ****p < 0.0001 by One-way ANOVA with Sidak’s multiple comparisons test. **F** Quantification of the rod index in DIV6 hippocampal neurons overexpressing PrP^C^ and pre-treated with 50 pM RAP103 for 24 h. Data represent mean±SEM (*n* = 3 independent samples/condition with ≥100 neurons/sample). ***p < 0.001, ****p < 0.0001 by One-way ANOVA with Tukey’s multiple comparisons test. **G** Representative images of differentiated SH-SY5Y cells expressing GFP or αSyn and immunostained for cofilin (red) and βIII-tubulin (blue). Scale bar: 10 μm. **H** qPCR results for *CCR5, PRNP and GAPDH* gene expression in differentiated SH-SY5Y cells. *ACTB* was used as a reference gene. Data represent mean ± SEM (*n* = 3 independent experiments). ***p < 0.001, ****p < 0.0001 by Student’s *t* test. **I** Schematic representation of the signaling pathway of αSyn-induced cofilin-actin rod formation in hippocampal neurons.

Having confirmed αSyn-induced rod formation in hippocampal neurons, we aimed at dissecting the molecular mechanism involved in this process. The major critical step for the generation of cofilin-actin rods is the localized dysregulation of cofilin activity via oxidation and dephosphorylation at the Ser3 residue. To validate that αSyn-induced rods were a result of cofilin activation, we performed co-transfection of the lentiviral plasmids with a cofilin phospho-mimetic inactive mutant (cofilin-S3E). This mutant is suggested to compete for binding to phosphatases, functioning as an inhibitor of the endogenous cofilin dephosphorylation [39]. In hippocampal neurons, we determined a 1.9-fold increase in rod index in DIV14 neurons expressing αSyn when compared to control cells (Fig. 4A and Supplementary Fig. S4A). Whereas cofilin-S3E co-expression had no effect on rod index in control cells, the expression of the cofilin mutant abolished αSyn-induced rods (Fig. 4A and Supplementary Fig. S4A), confirming that cofilin activation induced by αSyn mediates the formation of cofilin-actin rods.

In the AD context, Aβ-induced formation of rods was shown to occur via a PrP^C^-dependent pathway leading to NOX activation [23] and impacting on cofilin dysregulation. Thus, we addressed whether, similarly to Aβ, αSyn could act via PrP^C^, by overexpressing αSyn in hippocampal neurons from PrP^C^ KO mice. In WT neurons αSyn promoted a 2.3-fold increase in the percentage of neurons forming rods, whereas the protein was not able to induce rods in neurons from PrP^C^ KO mice (Fig. 4B). Similarly, the effect of αSyn PFFs on rods was abolished when using PrP^C^ KO neurons (Supplementary Fig. S4B). Additionally, to confirm the involvement of NOX in the formation of rods prompted by αSyn, we used neurons from p47 KO mice and observed that αSyn PFFs were not able to induce rod formation (Supplementary Fig. S4B).

In addition to the PrP^C^-NOX pathway, a recent report has shown that the chemokine receptors CXCR4 and CCR5 were involved in rod production in hippocampal neurons in response to gp120, an HIV-derived envelope protein, and Aβ dimers/trimers (Aβd/t) [22]. Interestingly, CCR5 has been extensively associated with memory and learning [24] which further supports testing its involvement in our settings. As such, we addressed whether the chemokine receptor CCR5 could also play a role in αSyn-induced hippocampal cofilin pathology. To test our hypothesis, we inhibited CCR5 by using an FDA-approved CCR5 antagonist, maraviroc. We observed a complete rescue of rod formation in αSyn-expressing hippocampal neurons when treated for 24 h with 50 nM maraviroc (Fig. 4C). To further validate our findings, we used RAP-103, a potent peptide antagonist of CCR5 signaling [40], with critical in vivo properties, namely rapid central nervous system (CNS) entry and stability with a long half-life in the brain. Confirming our findings with maraviroc, we observed that treatment with 50 pM of RAP-103 for 24 h completely abolished cofilin-actin rod formation in both the scenarios of αSyn overexpression (Fig. 4D, E) and of αSyn PFF addition (Supplementary Fig. S4C, D).

Since we found the involvement of both PrP^C^ and CCR5 in αSyn-induced hippocampal cofilin pathology, we questioned whether these players act on the same molecular pathway. To test this hypothesis, we cultured WT hippocampal neurons and overexpressed PrP^C^ which triggers rod formation as previously described (Fig. 4F) [23]. Treatment of PrP^C^ overexpressing neurons with 50 pM RAP-103 (>30 fold above the EC_50_ for inhibition of rods induced by Aβ in rodent neurons [41]) completely inhibited PrP^C^-induced rods (Fig. 4F), indicating that both receptors act on the same pathway to generate rods. Additional experiments reinforcing the requirement of both PrP^C^ and CCR5 to mediate αSyn-induced cofilin pathology were performed using the SH-SY5Y-based dopaminergic neuron-like cell model commonly used to mimic a PD-like phenotype in vitro [42]. Successful differentiation of SH-SY5Y cells was confirmed by immunostaining for the neuronal marker βIII-tubulin and TH, a marker for dopaminergic neurons (Supplementary Fig. S4E). In differentiated SH-SY5Y cells, αSyn overexpression (Supplementary Fig. S4F), did not prompt cofilin-actin rod formation (Fig. 4G). However, although we observed a significant expression of PrP^C^ in differentiated SH-SY5Y, the expression of CCR5 was nearly non-existent (Fig. 4H, Supplementary Fig. S4G), suggesting that, despite PrP^C^ expression, the lack of the chemokine receptor expression hinders cofilin-actin rod formation.

In summary, here we describe the interplay between PrP^C^ and CCR5 in the mechanism of generation of cofilin-actin rods prompted by αSyn (Fig. 4I). While the PrP^C^ receptor has been previously described as a molecular intervenient in the αSyn-induced synaptic dysfunction [33], our data suggest a novel role for CCR5 in that pathologic mechanism (Fig. 4I).

### CCR5 inhibition restores dendritic spine deficits triggered by αSyn, offering a promising therapeutic option for LBD

**Fig. 5:**
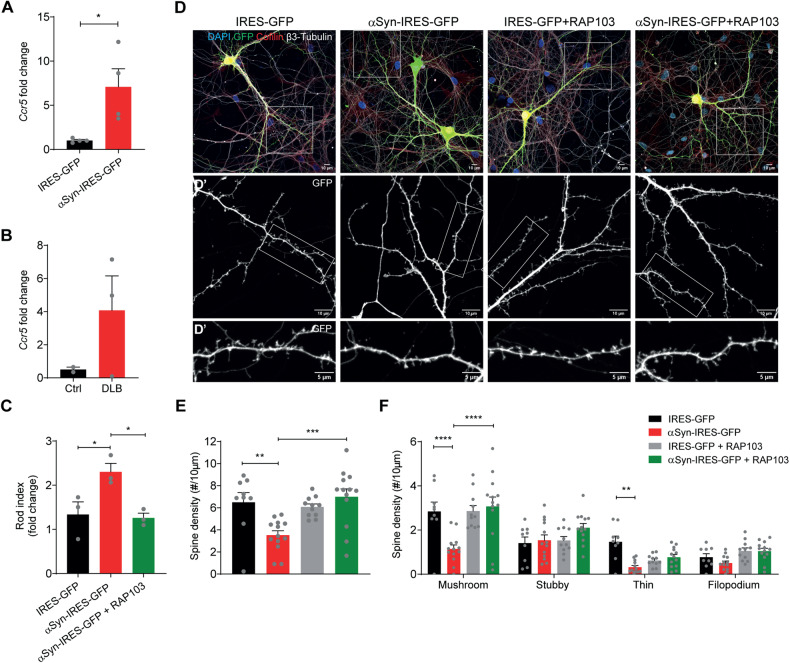
Blocking CCR5 rescues dendritic spine impairment induced by αSyn overexpression. **A** qPCR results for *Ccr5* gene expression in DIV14 hippocampal neurons expressing GFP or αSyn. Data shown as fold change in relation to the control sample. Data represent mean ± SEM (*n* = 4 independent experiments). *p < 0.05 by Student’s *t* test. **B** qPCR results for *CCR5* gene expression in Control and DLB patient samples. Data are shown as fold change in relation to control samples. *ACTB* was used as a reference gene. Data represent mean ± SEM (*n* = 2–3 cases/condition). **C** Rod index quantification of DIV14 hippocampal neurons expressing GFP or αSyn and pre-treated with RAP103 (50 pM) for 24 h. Data represent mean±SEM (*n* = 3 independent samples/condition with ≥100 neurons/sample). *p < 0.05 by One-way ANOVA with Tukey’s multiple comparison test. **D** Representative images of DIV14 hippocampal neurons expressing GFP or αSyn and pre-treated with 50 pM RAP-103 for 24 h, and immunostained for cofilin (red) and β3-tubulin (white). Scale bar: 10 μm. **D**’ Zoom-ins from **D**. GFP (white). Scale bar: 10 μm. **D**” Zoom-ins from **D**’. GFP (white). Scale bar: 5 μm. **E** Dendritic spine density relative to **D**. Data represent mean ± SEM (*n* = 9–13 dendrites/condition. Representative experiment). **p < 0.01, ***p < 0.001 by One-way ANOVA with Tukey’s multiple comparisons test. **F** Dendritic spine density by morphology relative to **D**. Data represent mean±SEM (*n* = 9–13 dendrites/condition. Representative experiment). **p < 0.01, ****p < 0.0001 by Two-way ANOVA with Tukey’s multiple comparison test.

After identifying CCR5 as a participant in the formation of cofilin-actin rods induced by αSyn, we assessed the levels of the receptor in our experimental conditions. For that, we measured the expression levels of *CCR5* by qPCR. We found them to be increased in αSyn-overexpressing hippocampal neurons (Fig. 5A), and in DLB patients when compared with control cases (Fig. 5B).

Considering that cofilin pathology contributes to neuronal dysfunction [21], we hypothesized that blocking CCR5 with the RAP-103 antagonist, which we showed to be a rod inhibitor, could have an impact on dendritic spine impairment caused by αSyn in hippocampal neurons. To test this effect, DIV14 hippocampal neurons overexpressing αSyn were treated with RAP-103 (50 pM) for 24 h. We observed that RAP-103 blockage of cofilin-actin rods in mature DIV14 αSyn-expressing hippocampal neurons (Fig. 5C) reverted dendritic spine defects (Fig. 5D–F). Specifically, RAP-103 completely rescued the alterations in spine number caused by αSyn overexpression, while not having an effect in control cells (Fig. 5D, E). Significantly, mushroom spine morphology which is most closely associated with mature synaptic function in memory and learning is the one most impacted by RAP-103 (Fig. 5F).

These results unravel CCR5 as a novel mediator of dendritic spine impairment induced by αSyn. Importantly, our work suggests CCR5 as a promising therapeutic target for LBD.

## Discussion

Hippocampal synaptic dysfunction in response to αSyn accumulation is a major cause of cognitive impairment in LBD. Here we demonstrate both in vitro and in vivo that high levels of αSyn, achieved either by αSyn overexpression or exogenous delivery of αSyn PFFs, in the rodent hippocampus induce cofilin pathology and disruption of dendritic spines, via a molecular mechanism involving both PrP^C^ and CCR5. Importantly, we show that the hippocampal cofilin pathology triggered by αSyn co-manifests with synaptic dysfunction and cognitive impairment in vivo by using the Thy1-aSyn mice, a suitable model to study cognitive deficits in the context of LBD [43]. Importantly, we determined that inhibition of the chemokine receptor CCR5, with a peptide antagonist with suitable features for future pre-clinical studies, completely rescues dendritic spine impairment caused by αSyn accumulation in hippocampal neurons.

Cofilin dysregulation was previously associated with αSyn pathology in primary cultures of hippocampal neurons, with αSyn-induced activation of the actin signaling pathway Rac1/PAK2/LIMK/cofilin-1 via GRP78 in hippocampal neurons, resulting in cofilin phosphorylation and inactivation, and consequent blockage of actin dynamics [44, 45]. Here, we present an opposite observation since αSyn overexpression triggers cofilin-actin rods in hippocampal neurons, which were reverted by using the phosphomimetic mutant cofilin-S3E, confirming αSyn-induced activation of cofilin to generate cofilin-actin rods. However, it is worth noting that either cofilin phosphorylation (inactivation) or its dephosphorylation (activation), and sequestering into rods reported here, would both result in a decline in cofilin-mediated actin dynamics that could impact synaptic plasticity. Additional work linking cofilin to αSyn demonstrated that cofilin immunostaining was increased in the striatum and cortex of αSyn A53T transgenic mice and brains from PD patients, correlating with high levels of αSyn pS129 [46]. That work also demonstrated that cofilin accelerates αSyn aggregation and forms highly toxic mixed cofilin-αSyn fibrils which when injected in the striatum of WT mice induce higher dopaminergic pathology than pure αSyn fibrils injection [46, 47]. In our experiments, we were not able to colocalize cofilin-actin rods with αSyn, as distinct immunostaining protocols are required to visualize rods and αSyn. Although in the context of cofilin being a promoter of αSyn aggregation, and not cofilin-actin rod pathology, the referred work supports our data on cofilin being related to αSyn-induced neurodegeneration. Nevertheless, we focused on hippocampal pathology and we did not observe cofilin pathology in the *substantia nigra* of Thy1-aSyn mice so we cannot link, based on our data, cofilin-actin rod formation to dopaminergic dysfunction.

αSyn-induced rods occur by a pathway involving PrP^C^, NOX, and CCR5 (Fig. 4I), similar to what was reported for Aβ and more recently for the HIV viral envelope glycoprotein gp120 [22, 23]. PrP^C^, NOX, and chemokine receptors are believed to be concentrated in specific areas of the cell membrane namely in lipid rafts [48, 49]. Lipid rafts act as a platform that helps bring signaling proteins together and increases the possibility of interactions between the receptors [48, 49]. One important consequence of this molecular organization is the potential crosstalk between the signaling pathways we have identified in αSyn-mediated hippocampal pathology. We suggest that PrP^C^ might serve as a co-receptor facilitating αSyn interaction with chemokine receptors to control synapse structure and function. Supporting this idea, we demonstrate that CCR5 blockage inhibits PrP^C^-induced rods and that neuronal cells expressing PrP^C^ but with residual CCR5 expression do not form cofilin-actin rods.

Overexpression of cofilin in cultured hippocampal neurons results in the formation of rod-shaped structures that negatively impact synaptic function [21]. We confirmed that cofilin pathology is involved in αSyn-induced spine impairment. It is worth noting that αSyn overexpression is a pathologic stimulus, unlike the overexpression of cofilin, which further emphasizes the importance of our findings. Cofilin pathology might have consequences on synaptic function through either physical blockage of vesicle transport or sequestration of cofilin, depleting it from synaptic structures and impacting in actin dynamics and function of these structures [21, 50].

PrP^C^ was previously described to interact with αSyn, mediating hippocampal synaptic impairment and cognitive deficits in PD [33]. Considering the inhibitory effect of PrP^C^ knockdown on αSyn-induced rods, our data includes cofilin pathology as a new intermediate on the mechanism of αSyn-PrP^C^ induction of synaptic and cognitive dysfunction. Importantly, one of the major findings of the current work showed that blocking CCR5 is sufficient to rescue completely dendritic spine impairment triggered by αSyn. This finding is in accordance with studies showing that CCR5 acts as a suppressor for neuronal plasticity and consequent learning and memory impairments [24]. Specifically, it was shown that CCR5 KO mice present enhanced LTP and hippocampal-dependent memory and that the overexpression of CCR5 causes memory deficits [24, 25]. Additionally, in mouse models of cortical stroke and traumatic brain injury, where neuronal dysfunction was observed, CCR5 was found upregulated and its knockdown or pharmacologic inhibition improved motor and cognitive function, an effect suggested to be derived from the preservation of dendritic spines [51].

Focusing on the link between CCR5 and cofilin activity, some studies have shown cofilin activation upon HIV gp120 binding to chemokine receptors in blood-resting CD4 T cells during infection [52]. HIV binding to chemokine receptors triggers cycles of cofilin activation, leading to increased cortical actin dynamics facilitating virus entry [52–55]. Together, these studies suggest a CCR5 regulation of cofilin activity, which further supports our findings.

To target CCR5 we used RAP-103, which is an orally available and shorter analog of the clinically validated octapeptide D-ala1-peptide T-amide (DAPTA), a gp120-derived CCR5 inhibitor that was shown to protect spines in vivo [56]. Considering our findings, RAP-103 was proven to be more potent than the FDA-approved CCR5 antagonist maraviroc, by at least a 1000-fold increase in rod inhibition [41]. RAP-103 is safe with rapid blood-brain barrier penetration and entry into the CNS, and easy oral administration, making it suitable for further development in dementias.

A major therapeutic gap in LBD is the absence of approved drugs that have an effect on disease progression. This is a large unmet need for millions of people living with LBD. Off-label drugs include cholinesterase inhibitors and memantine for cognitive symptoms [57, 58]; dopaminergic medications for motor symptoms [59]; modafinil and armodafinil for cognitive fluctuations and attention [60, 61]; antidepressants, atypical antipsychotics, antiepileptics, and prazosin for behavioral symptoms [61]. As such, the development of new therapies targeting dementia in the context of synucleinopathies is imperative. In this respect, our work proposes targeting CCR5 by using a promising molecule such as RAP-103 in in vivo models of LBD. Supporting our hypothesis, we validated the presence of cofilin-actin rods and increased expression of CCR5 in the hippocampal region of DLB patients when compared to age-matched controls. To further consolidate these findings, it will be important to examine a larger number of human cases.

Taking all this into consideration, we unraveled a role for chemokine receptors in cofilin pathology and synaptic dysfunction, of which PrP^C^ serves as a co-receptor, and we propose that targeting CCR5 constitutes a promising therapeutic approach, not only for LBD but also for AD or HAND (HIV-associated neurocognitive disorders), where rods, synaptic dysfunction and cognitive impairment were reported [22, 31, 62, 63]. Overall, our results strengthen the link between αSyn-induced hippocampal synaptic dysfunction and cognitive deficits in LBD and identify cofilin and CCR5 as novel pathologic players.

## Materials and methods

### Human samples

Brain samples were obtained from the Portuguese Brain Bank (PBB) through appropriate consent procedures for the collection and use of human brain tissues. According to PBB tissue donation policy, appropriate consent was obtained from all subjects for brain donation. The PBB scientific committee reviewed and approved the tissue request for research. According to PBB protocol, half of the brain is dissected fresh upon collection and regions of interest flash frozen. Frozen sections of the hippocampal region were used for the qPCR analysis and hippocampal paraffin sections were cut at 6 μm for immunohistochemistry studies. The summary of clinical information of human samples can be found in Supplementary Table 1.

### Animals

All animals were handled according to EU Directive 2010/63/EU and decree-law n° 113-2013. The protocols described in this work have been approved by the i3S Ethical Committee, by the Portuguese Veterinarian Board, and by the Institutional Animal Care and Use Committee of Colorado State University (protocols KP1023 and KP1412). Transgenic mice overexpressing human αSyn under the neuronal Thy-1 promoter (Thy1-aSyn mice, Line 61 developed on a C57BL/6/DBA2 background) [34] were kindly provided by the University of California San Diego. The colony background was maintained by breeding mutant females with WT C57BL/6-DBA/2 males. Since the transgene insertion was on the X chromosome and there is random inactivation of the X chromosome, only male littermates were used in the experiments. PrP^C^ null mice (PrP-/-) TALEN [64] were generously provided by Mark Zabel, Prion Research Center, Colorado State University. Time pregnant wild-type female Wistar rats (E18) or C57BL/6 mice (E16.5) were used for the dissociated hippocampal neuronal cultures. For αSyn PFFs stereotactic injection, we used C57BL/6 mice in which a microinjection syringe was inserted to target the *striatum* unilaterally (A: 1.0, L: 1.9, D: 3.0). Each injection delivered 5 µL of 5 μg/μL PFFs or, for controls, 5 µL of saline. Post-surgical incisional pain was treated with bupivacaine and buprenorphine.

### Behavioral tests

6-month-old Thy1-aSyn mice were used in behavioral tests and thereafter killed and their brains collected.

#### Morris water maze

Spatial learning was assessed by the hidden-platform MWM test. A circular pool (diameter 111 cm) was used and filled with water at 21 ± 1 °C. The pool was theoretically divided into four quadrants and eight start positions were defined at equal distances to the center. An escape platform (10 × 10 cm) was placed 0.5 cm below the water line. In the first two days, during the cued learning, mice were trained to find the hidden platform which had a visual clue. Animals were subjected to four swimming trials which had different start and goal positions. In the next 5 days, during the learning phase, mice were trained to find the hidden platform which was in the same location during the entire learning phase. Every day mice were subjected to four swimming trials, each trial starting at one of the four different pool locations, and the latency to find the platform was scored. If mice failed to find the platform within 1 min they were guided to the platform. In either case, mice were allowed to stay on the platform for 15 s. On day 8, in the probe day, the platform was removed from the pool and the mice were allowed to swim for 30 sec. Swimming tracks were recorded and analysis of the total distance, distance in target, and target crossings were obtained with The Smart v3.0, Panlab, Barcelona, Spain.

#### Novel object recognition

Animals that have been previously subjected to habituation were subsequently subjected to the NOR test to evaluate recognition memory. In the arena, animals were exposed to two identical objects for 10 min for familiarization. In the test phase, 4 h later, one of the familiar objects was replaced by a new object from the same material, weight, and height, but with a different color and shape, and the animal was allowed to explore for 3 min. Exploration was considered when the mouse’s nose touched the object or when the nose is directed to the object from a distance less than 2 cm. Mouse behaviors were recorded and analyzed by the software The Observer XT v7.0, Noldus, Netherlands. The discrimination index was calculated by DI = (*T*_N_ − *T*_F_)/(*T*_N_ + *T*_F_) in which *T*_N_ is the time exploring the novel object and *T*_F_ is the time spent exploring the familiar object. Animals with a total exploration time (novel + familiar) <10 seconds were excluded from the analysis of the discrimination index [65].

### Plasmids and viral vectors

IRES-GFP and WT αSyn-IRES-GFP lentiviral plasmids were previously described [66]. Briefly, full-length human WT αSyn cDNA was subcloned into the pWPI vector (second-generation bicistronic lentiviral vector, Tronolab, Switzerland), under the chicken/β-actin (CBA) promoter. A pWPI vector containing only IRES-GFP was used as control. pmRFP-N1 and pmRFP-N1-Cofilin-S3E plasmids were described previously [67]. Briefly, pseudo-phosphorylated human cofilin-1 (S3E) was cloned into the pmRFP-N1 backbone vector, under the CMV promoter. An empty pmRFP-N1 vector was used as control. CMV-hPRNP-mCherry and CMV-hCCR5-mCherry plasmids were customized and purchased from VectorBuilder.

### Lentiviruses production and titration

Lentivirus production was performed as previously described [68]. Briefly, HEK293T cells were transfected, using Lipofectamine 2000 (ThermoFisher Scientific, 11668030), with the DNA complexes containing the plasmid of interest and the packaging plasmids (psPAX2 and VSV-G), for 5 h at 37 °C/5% CO_2_. After the incubation, the medium was replaced with DMEM (VWR, 733-1695) supplemented with 10% fetal bovine serum (FBS, Biowest, BWSTS181BH-500) and 1% penicillin-streptomycin (P/S, 100 U/mL, ThermoFisher Scientific, 15140122). After 48 h, the lentivirus-containing supernatants were recovered, centrifuged for 10 min at 500 g and filtered through a 0.45 μm filter (Enzifarma). The filtered supernatants were concentrated using a centricon (GE Healthcare Life Sciences), aliquoted and stored at −80 °C. For virus titration, HEK293T cells were infected with different volumes of lentivirus. After 2 days, cells were resuspended in PBS and the total number of transduced cells was analyzed by Flow Cytometry using FACS Accuri (BD Biosciences). The lentiviral transfection units (TU) per μL were determined by the following equation: TU/μL = (number of plated cells x % of infected cells (GFP positive)) / volume of viral particles added (μL).

### Dissociated hippocampal neuron cultures and transduction

The hippocampus was dissected from E18 rat embryos (WT) or E16.5 mouse embryos (WT, PrP^C^ KO or p47 KO), digested with 0.06% trypsin (Sigma-Aldrich, T4799) in Hanks’ balanced salt solution (HBSS, Sigma, H9394) for 15 min at 37 °C. Following digestion, neurons were dissociated by gentle trituration and resuspended in neurobasal medium (Invitrogen, 21103049) supplemented with 2% N21-MAX (R&D Systems, AR008), 1% P/S and 2.5 mM L-Glutamine (Lonza, 17-605E). Cells were then counted and plated at a density of 15,000 cells/coverslip in a 24-well plate for immunostaining analysis, or at a density of 200,000 cells/well in a 6-well plate for western blot analysis and qPCR. Coverslips and plates were precoated with 20 µg/mL poly-D-lysine (Sigma, P0899). For neuronal transduction, DIV3 hippocampal neurons were treated with 1 µM of (+)-MK-801 hydrogen maleate (Sigma, M107) for 30 min at 37 °C to reduce spontaneous rod formation. At DIV4, neurons were infected either with WT αSyn-IRES-GFP or IRES-GFP lentiviruses (1 TU/cell). DIV7 or DIV14 neurons were fixed for imaging or lysed for cell extracts.

### αSyn pre-formed fibrils

For PFF preparation, human αSyn monomer protein was purchased from Proteos (RP-003). PFFs were prepared according to the protocol established by the Michael J Fox Foundation for Parkinson’s Research [69]. Briefly, αSyn monomers were diluted to 5 mg/mL into 0.01 M phosphate-buffered saline (PBS) containing 0.03% sodium azide to prevent bacterial growth. Monomers were shaken for 7 days at 1000 rpm at 37 °C in an orbital shaker to induce the formation of fibrils (PFFs). Single-use aliquots were rapidly frozen and stored at −80 °C. Immediately before use, frozen aliquots were thawed, diluted in PBS to 0.1 mg/mL and bath sonicated at room temperature for 5 min. For DIV7 experiments, 1 µg/mL of PFFs were added to hippocampal neurons for 3 h, 6 h, 12 h, 18 h, or 24 h before fixation. In the experiments with drugs, DIV7 hippocampal neurons were treated with 1 µg/mL of PFFs with the drugs or vehicles for 24 h before fixation. For DIV14 experiments, 150 ng/mL or 1 μg/mL of PFFs were added at DIV7 or DIV13, respectively.

### Hippocampal neuron transfection

DIV3 hippocampal neurons were treated with 1 μM of (+)-MK-801 hydrogen maleate (Sigma, M107) for 30 min at 37 °C to reduce spontaneous rod formation. At DIV12 neurons were transfected using the calcium phosphate co-precipitation method with the constructs: αSyn-IRES-GFP, IRES-GFP, pmRFP-N1-Cofilin-S3E, and pmRFP-N1. Briefly, a maximum amount of 2 μg of DNA (single or mixture) was diluted in Tris-EDTA (TE) pH 7.3 and mixed with HEPES calcium chloride (2.5 M CaCl_2_ in 10 mM of HEPES pH 7.2). This mixture was added to 2x HEBS (270 mM NaCl, 10 mM KCl, 1.4 mM Na_2_HPO_4_, 11 mM Dextrose, 42 mM HEPES pH7.2) and the precipitate was allowed to develop during 30 min at RT in the dark, gently mixing every 5 min. For cell transfection culture medium was removed and saved while neurons were incubated with neurobasal medium, without supplements, and the precipitates were added dropwise to each well. Precipitates were incubated with cells for 45 min at 37 °C/5% CO_2_. The precipitate solution was then removed and neurons were washed with acidic neurobasal medium (equilibrated at 10% CO_2_) for 20 min at 37 °C/5% CO_2_. Lastly, the medium was replaced with the saved culture medium. Neurons were analyzed at DIV14, 48 h after transfection.

### Drug treatments

For RAP-103 or maraviroc treatment, DIV6 or DIV13 hippocampal neurons either transduced, transfected, or treated with αSyn PFFs, were treated with RAP-103 ((all-D-TTNYT) CCR5 antagonist, Creative BioPeptides, Inc.) at 50 pM diluted in water, or maraviroc (Sigma, PZ0002) at 50 nM diluted in PBS. Treated neurons were analyzed at DIV7 or DIV14.

### SH-SY5Y cell differentiation and treatments

SH-SY5Y cells were purchased from Sigma (94030304). SH-SY5Y cells were cultured in DMEM/F12 (Sigma-Aldrich, D6434) supplemented with 10% FBS and 1% P/S and frequently tested for mycoplasma contamination. For neuronal differentiation, the culture medium was changed to DMEM/F12, 2% B27 (Invitrogen, 17504044), 1% P/S, 10 µM all-trans-retinoic acid (Fisher Scientific, 10552611). After 2 days the medium was renewed with fresh all-trans-retinoic acid. At DIV6 the medium was replaced with DMEM/F12, 2% B27, 1% P/S, and 50 ng/ml BDNF (Peprotech EC, 450-02) or 8 nM phorbol 12-myristate 13-acetate (PMA, Sigma-Aldrich, P8139). Differentiated cells were transduced with WT αSyn-IRES-GFP or IRES-GFP lentiviruses (1 TU/cell) and after 3 days cells were fixed for imaging. For expression analysis, RNA was extracted from differentiated cells non-transfected, or transfected with CMV-hPRNP-mCherry or CMV-hCCR5-mCherry, cDNA was then synthesized and gene expression analysis performed by qPCR.

### Immunocytochemistry

For cofilin-actin rod staining, neurons were permeabilized with 100% methanol at -20 °C for 3 min at RT and blocked with 2.5% normal serum from donkey (Jackson ImmunoReasearch, 017-000-121) or goat (Sigma, 19H092) in 1% BSA/PBS for 1 h at RT. Incubation with primary antibodies: rabbit anti-T-Cofilin 1:2000 (Bamburg lab, 1439 or Cell Signaling, 5175) and mouse anti-β3-tubulin 1:2000 (Promega, G7121) diluted in 1% BSA/PBS, was performed overnight at 4 °C. For TH staining, a similar protocol was followed, with incubation of primary antibodies as follows: chicken anti-TH 1:1000 (Abcam, ab76442) and mouse anti-β3-tubulin 1:2000 (Promega, G7121) diluted in 1% BSA/PBS and incubated overnight at 4 °C. For αSyn immunostaining, neurons were permeabilized with 2.5% triton X-100 in PBS for 20 min at RT and blocked with 5% normal donkey serum in 1% BSA/PBS for 1 h at RT. Subsequently, neurons were incubated with primary antibodies: mouse anti-αSyn 1:1000 (BD Biosciences, 610787) and rabbit anti αSyn pS129 1:1000 (Abcam, ab51253) diluted in 1% BSA/PBS and incubated overnight at 4 °C. For all immunolabelings, after washing off primary antibodies, cells were incubated with secondary antibodies: donkey anti-mouse-Alexa Fluor 568 1:1000 (Invitrogen, A10037), donkey anti-chicken-Alexa Fluor 568 1:1000 (Invitrogen, A78950), donkey anti-rabbit–Alexa Fluor 568 1:1000 (Invitrogen, A10042), donkey anti-mouse-Alexa Fluor 647 1:1000 (Invitrogen, A31571) or donkey anti-rabbit-Alexa Fluor 647 1:1000 (Jackson ImmunoResearch, 711-605-152), diluted in 1% BSA/PBS. Coverslips were mounted in Fluoromount-G (SouthernBiotech, 0100-01) or ProLong Diamond Antifade (ThermoFisher P36961).

### Immunohistochemistry

For mouse brain immunostaining analysis, 6-months post-PFFs injection mice or 6-month-old Thy1aSyn mice were perfused with PBS for 5 min followed by 4% paraformaldehyde (PFA, pH 7.4) in PBS. Brains were incubated in 4% PFA for 24 h and then in 30% sucrose. Brain tissues were embedded in Optimum Cutting Temperature (OCT) compound (ThermoFisher Scientific), frozen, and sectioned coronally (Cryostat Leica CM3050S) at 30 μm. Sections were then permeabilized with 100% methanol at -20 °C for 5 min at RT. For human brains, 6 μm paraffin sections were de-waxed and re-hydrated followed by an antigen retrieval step where slides were microwaved in water for 8 min. For cofilin-actin rods staining, sections were blocked with 5% normal donkey serum in PBS for 1 h at RT, followed by incubation overnight at 4 °C with primary antibodies: rabbit anti-T-cofilin 1:1000 and chicken anti-TH 1:1000 (Abcam, ab76442) and subsequent washing and incubation with secondary antibodies: donkey anti-rabbit–Alexa Fluor 568 1:500 with donkey anti-chicken–Alexa Fluor 488 diluted in 1% BSA/PBS. Brain sections were then washed and rinsed in 70% ethanol and incubated with 0.1% Sudan Black in 70% ethanol for 10 min at RT. Sections were washed, incubated with DAPI (Bio-Rad, 1351303) for 10 min and mounted in ibidi mounting medium (ibidi, 50001). For staining of αSyn pS129, mouse brain sections were washed with 0.3% triton X-100 in PBS and blocked with 1% normal donkey serum in 0.3% triton X-100 in PBS for 1 h at RT, followed by incubation with primary antibody: rabbit anti-αSyn pS129 (Abcam, ab51253) 1:5000 (Thy1-aSyn sections) or 1:500 (PFF-injected sections) diluted in blocking buffer and incubated 48 h (Thy1-aSyn sections) or 24 h (PFF-injected sections) at 4 °C. After washing the primary antibodies, sections were incubated with the secondary antibody donkey anti-rabbit–Alexa Fluor 568/594 1:1000 for 2 h/1 h at RT. After the secondary antibody, sections were washed, incubated with DAPI for 10 min and mounted in ibidi mounting medium. For PSD-95 staining, sections were permeabilized and blocked with 10% FBS with 0.2% Triton in PBS for 1 h at RT and then incubated with primary antibody mouse anti-PSD-95 1:500 (ThermoFisher, MA1-045) diluted in blocking buffer and incubated 48 h at 4 °C. After washing the primary antibody, sections were incubated with the secondary antibody donkey anti-mouse–Alexa Fluor 568 1:500 for 2 h at RT, then washed and rinsed in 70% ethanol and incubated with 0.1% Sudan Black in 70% ethanol for 10 min at RT. Sections were washed, incubated with DAPI for 10 min and mounted in ibidi mounting medium.

### Western blot

For western blot analysis, 6-month-old mice were perfused with PBS for 5 min, and the hippocampus dissected and quickly frozen in dry ice. Frozen brains were incubated with RIPA buffer (1% Triton X-100, 0.1% SDS, 140 mM NaCl, 1x TE pH 8, 1x protease inhibitor cocktail and 1 mM sodium orthovanadate), sonicated (2 × 10 cycles, Output Power 50 Watts, Branson sonifier 250) and cleared by centrifugation at 15,000 rpm for 5 min at 4 °C. Neuronal cell lysates were sonicated (2 × 10 cycles, Output Power 50 Watts, Branson sonifier 250) and cleared by centrifugation at 15,000 rpm for 5 min at 4 °C. Protein extracts (25 μg or 5 μg) were separated under denaturing conditions in 12% SDS-PAGE gels and transferred to nitrocellulose membranes (0.45 μm GE HealthCare). Membranes were blocked with 5% milk (Sigma-Aldrich) in TBS-T or 5% BSA (NZYTech) in TBS-T for 1 h at RT. Membranes were probed overnight at 4 °C with the following primary antibodies: mouse anti-αSyn 1:1000 (BD Biosciences, 610787), rabbit anti-αSyn pS129 1:500 (Abcam, ab51253), rabbit anti-T-Cofilin 1:1000 (Bamburg lab, 1439 or Cell Signaling, 5175), mouse anti-T-Cofilin 1:500 (Abcam, ab54532), rabbit anti-P-Cofilin Ser3 1:1000 (Cell Signaling, 3311), mouse anti-PSD-95 1:2000 (ThermoFisher Scientific, MA1-046), rabbit anti-Vinculin 3:10000 (ThermoFisher Scientific, 700062), chicken anti-GFP 1:3500 (Aves Labs GFP-1020) and mouse anti-GAPDH 1:1000 (Santa Cruz, sc-166574) diluted in 5% milk/TBS-T or 5% BSA/TBS-T. After washing membranes were incubated for 1 h at RT with the secondary antibodies: anti-mouse IgG-HRP 1:10000 (Jackson Research, 115-035-003), anti-rabbit IgG-HRP 1:10000 (Jackson Research, 111-035-003) or anti-chicken IgG-HRP 1:10000 (Jackson Research, 703-545-155) diluted in 5% milk/TBS-T or 5% BSA/TBS-T. Immunodetection was performed by chemiluminescence using ECL (Millipore, WBLUR0500). Quantitative analyses were performed with the Quantity One software, Image Lab software or Fiji software.

### RNA isolation and real-time RT-PCR

Total RNA from DIV14 transduced hippocampal neurons, differentiated SH-SY5Y cells and human brain sections were extracted using NZY total RNA isolation kit (NZYTech, MB13402). An average of 1.5 μg of total RNA were used to synthesize first-strand cDNA (NZY First-Strand cDNA Synthesis Kit, MB125). SYBR-green quantitative PCR (CFX384 Touch™ Real-Time PCR Detection System, Bio-rad) was performed using specific primers. Rat hippocampal neurons: Ccr5, sense primer: CGCTGTAGGAATGAGAAGAAGAGG, antisense primer: AAGGTGGTCAGGAGGAGGA; b-actin, sense primer: GCCCCTCTGAACCCTAAG, antisense primer: ACAACACAGCCTGGATGG. The fold change in gene expression was calculated using the ΔΔCt relative expression method (Livak method) and primers for β-actin were used as the endogenous control and calculated separately for each sample and respective condition. SH-SY5Y and human brains: CCR5, sense primer: GACATCTACCTGCTCAACCT, antisense primer: AGATTCCAGAGAAGAAGCCTAT; PrP^C^, sense primer: GTGACTATGAGGACCGTTACT, antisense primer: CGTGTGCTGCTTGATTGT; GAPDH, sense primer: CGGATTTGGTCGTATTGG, antisense primer: GGTGGAATCATATTGGAACA; β-actin, sense primer: ACAGAGCCTCGCCTTTGCCG, antisense primer: CACCATCACGCCCTGGTGC. The fold change in gene expression was calculated using the ΔΔCt relative expression method (Livak method) and primers for β-actin or GAPDH were used as the endogenous control and calculated separately for each sample and respective condition.

### Imaging and quantifications

Hippocampal neurons cultured for 7 days and immunostained for cofilin were assessed for the presence of cofilin-actin rods with an upright epifluorescence microscope (Zeiss Axio Imager Z1, Carl Zeiss) at 40x magnification. The percentage of neurons with cofilin-actin rods was manually scored and plotted. Imaging of cofilin in DIV7 PFF-induced rods was performed on a Keyence Fluorescence Microscope with a 20x objective. DIV14 hippocampal neurons immunostained for cofilin and β3 tubulin were imaged in an automated fluorescence widefield high-content screening microscope (IN Cell Analyzer 2000, GE Healthcare) at 40x magnification. Images were analyzed using Fiji software and the ratio between the number of rods and the total number of neurons analyzed was calculated and plotted as Rod index. For spine density quantification, dendritic spines visualized by GFP signal were imaged in a laser scanning Confocal Microscope Leica SP8, using the 63x glycerol objective. Dendritic length and spine number and morphology were quantified using the semi-automatic NeuronStudio software. Dendritic spine morphology was defined as mushroom spines (small neck and large head), thin spines (long neck and small head), stubby spines (head without a defined neck) and filopodium spines (long neck without defined head). Brain sections stained for cofilin were imaged in an automated fluorescence widefield high-content screening microscope (IN Cell Analyzer 2000, GE Healthcare) at 20x magnification. Images were stitched and the brain regions of interest analyzed using Fiji software and the results plotted as the number of rods per area. Brain sections stained for PSD-95 were imaged in a laser scanning Confocal Microscope Leica SP8, using the 63x glycerol objective. PSD-95 puncta were quantified using the Puncta Analyser plug-in in Fiji software.

### Statistical analysis

All measurements were performed with the researcher blinded to the experimental condition. Data are shown as mean ± SEM. We conducted the Shapiro-Wilk test to assess the normality of the data and established that all data followed a normal distribution. Unpaired t-tests were used for comparing differences between two groups, while one-way ANOVA or two-way ANOVA followed by Tukey’s multiple comparisons test, by Sidak’s multiple comparisons or by Dunnett’s multiple comparison’s test was applied to identify significant differences among multiple groups. For the in vivo experiments the sample size was chosen based on previous research with the Thy1aSyn mouse model [33] and with the αSyn PFFs injected mouse model [36]. For the in vitro analysis all the experiments were performed at least three times. Statistical significance was determined using the GraphPad Prism Software version 8 being significance determined by *p < 0.05, **p < 0.01, **p < 0.001 and ****p < 0.0001. Statistical tests and sample sizes are indicated in each figure legend.

### Supplementary information

Suplementary Information

Original Data File

## Data Availability

All data generated and analyzed during this study are included in this published article (and its supplementary information files) and available from the corresponding author upon reasonable request.
