# Supplementary material for: α-Synuclein triggers cofilin pathology and dendritic spine impairment via a PrPC-CCR5 dependent pathway
Source: Cell Death Dis. 2024 Apr 13;15(4):264. doi: 10.1038/s41419-024-06630-9 (PMC11016063; doi:10.1038/s41419-024-06630-9)
Supplement: Supplementary file 1 — Suplementary Information [file 41419_2024_6630_MOESM1_ESM.docx]

**α-Synuclein triggers cofilin pathology and dendritic spine impairment *via* a CCR5-PrP^c^ dependent pathway**

This file contains:

- Supplementary Table

- Supplementary Figures

- Supplementary Figure Legends

**Supplementary Table 1- Clinical information of human samples**

| Case number | Sex | Age at onset | Age at Death | PMD | Cause of death | Neuropathological Diagnoses |
| --- | --- | --- | --- | --- | --- | --- |
| BC0172 | Female |  | 72 | 19h | Colon adenocacinoma | control |
| BC0179 | Male |  | 71 | 16.5 | Lung carcinoma | control |
| BC0161 | Male | 61 | 64 | 19h | Pneumonia | Dementia with Lewy Bodies (McKeith, 2017)  - Diffuse neocortical  LB pathology  - Alzheimer´s disease neuropathology change: Braak stage IV; Thal phase 3; CERAD “moderate” |
| BC0176 | Female | 71 | 78 | 45.5h | Cachexia associated to end-stage dementia | Dementia with Lewy Bodies (McKeith, 2017)  - Diffuse neocortical  LB pathology  - Alzheimer´s disease neuropathology change: Braak stage IV; Thal phase 3; CERAD “moderate” |
| BC0177 | Male | 77 | 81 | 40h | Cachexia associated to end-stage dementia | Dementia with Lewy Bodies (McKeith, 2017)  Secondary diagnosis  - PART, definitive  (Braak stage II)  - Mild small vessel disease |

**
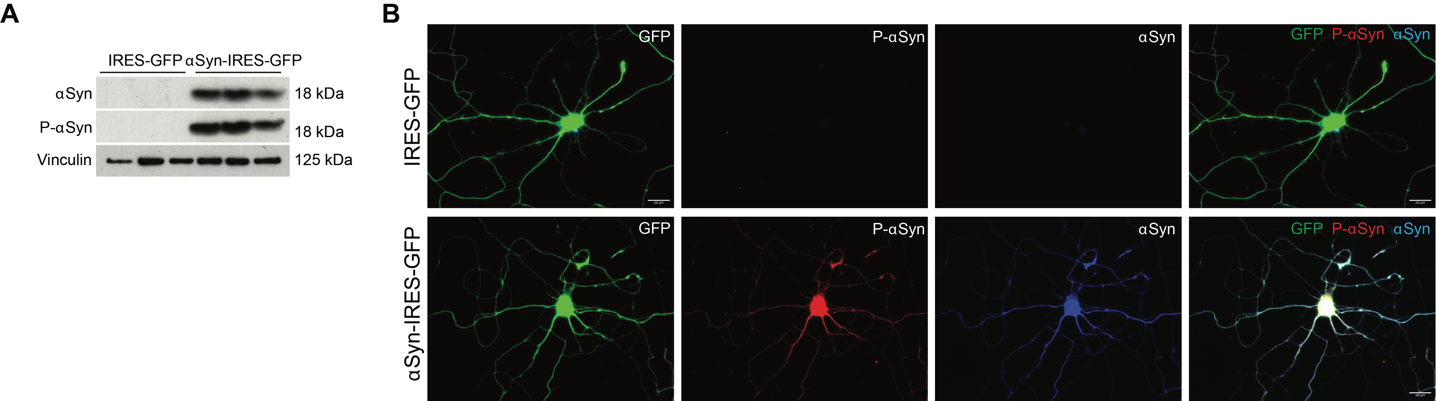
**

**Supplementary Fig. S1 - Overexpression of αSyn in DIV7 hippocampal neurons. A** Representative western blot of αSyn and αSyn pS129 levels in DIV7 hippocampal neurons expressing GFP or αSyn. Vinculin was used as loading control. Data represent mean±SEM (n=3 independent samples/condition). **B** Representative images of DIV7 hippocampal neurons expressing GFP or αSyn immunostained with αSyn pS129 (red) and αSyn (blue). Scale bar: 20 μm.


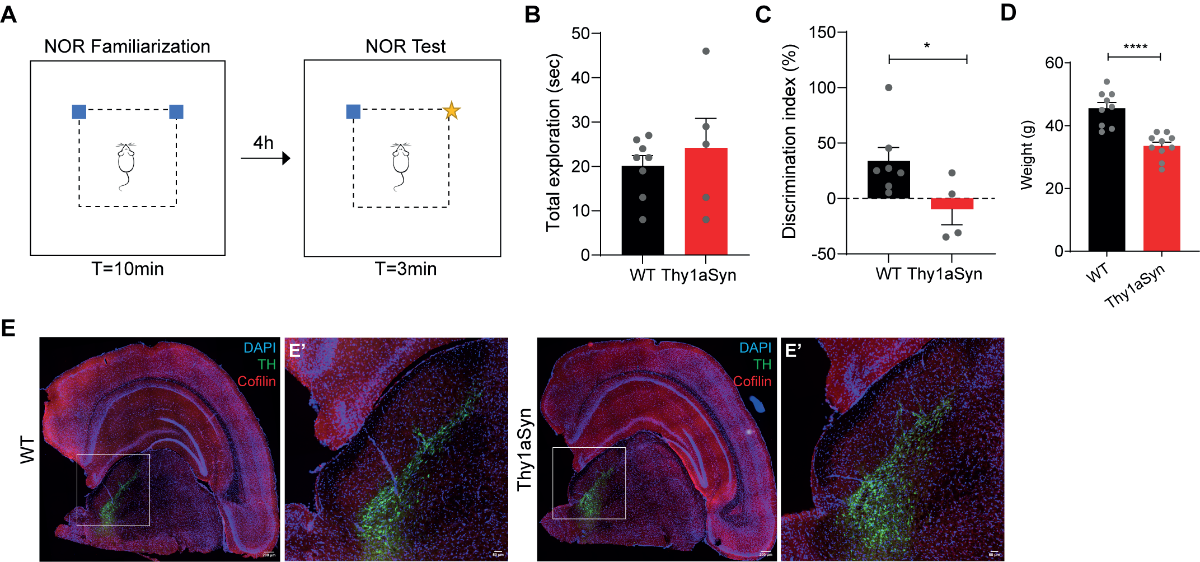


**Supplementary Fig. S2 - Thy1-aSyn mice show decreased body weight, alterations in the NOR test and no cofilin pathology in the substantia nigra**. **A-C** Novel object recognition (NOR) test in 6-month-old animals. **A** Schematic representation of the NOR test. **B** Total exploration time of the familiar and new objects in the NOR test. **C** Percentage of discrimination index in the NOR test. Data represent mean±SEM (n=4-7 animals/condition). *p<0.05 by Student’s t test. **D** Weight of 6-month-old WT and Thy1-aSyn mice. Data represent mean±SEM (n=9-10 animals/condition). ****p<0.0001 by Student’s t test. **E** Representative images of brain sections from 6-month-old WT and Thy1-aSyn mice immunostained for TH (green) and cofilin (red). DAPI (blue). Scale bar: 200 μm. **E’** Zoom-ins of the substantia nigra region from E. Scale bar: 50 μm.

**
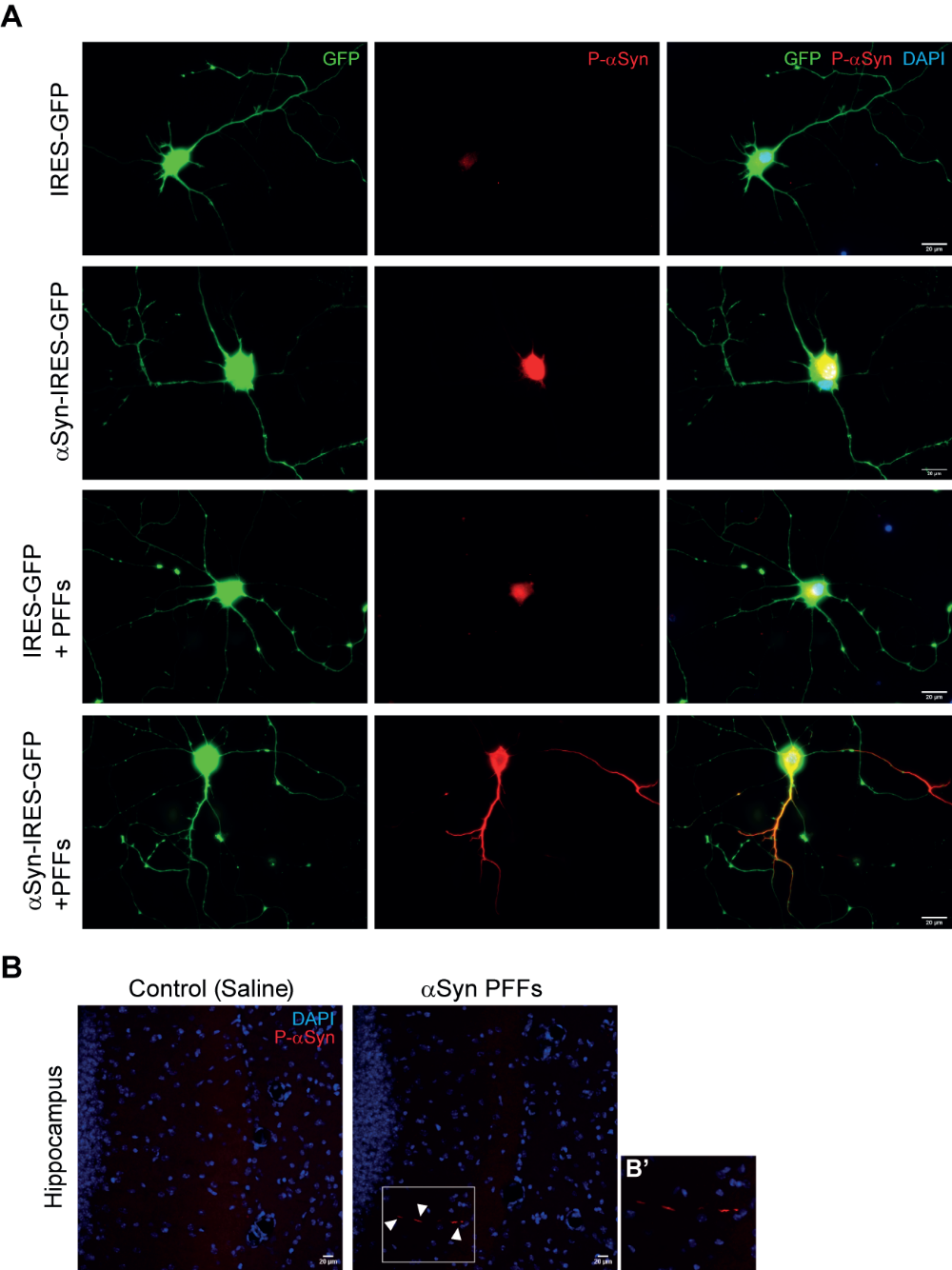
**

**Supplementary Fig. S3 - αSyn PFFs trigger αSyn aggregation in primary hippocampal neurons and *in vivo* in hippocampus.**

**A** Representative images of DIV14 hippocampal neurons expressing GFP or αSyn, untreated or pre-treated at DIV7 with αSyn PFFs and immunostained for αSyn pS129 (red). Scale bar: 20 μm. **B** Representative images of hippocampus brain sections from control (Saline) and αSyn PFFs injected WT mice 6 months post-injection immunostained for P-αSyn 129 (red). DAPI (blue). Arrowheads and inset indicate P-αSyn 129 positive aggregates. Scale bar: 20 μm. **B’** Zoom-ins from B.

**
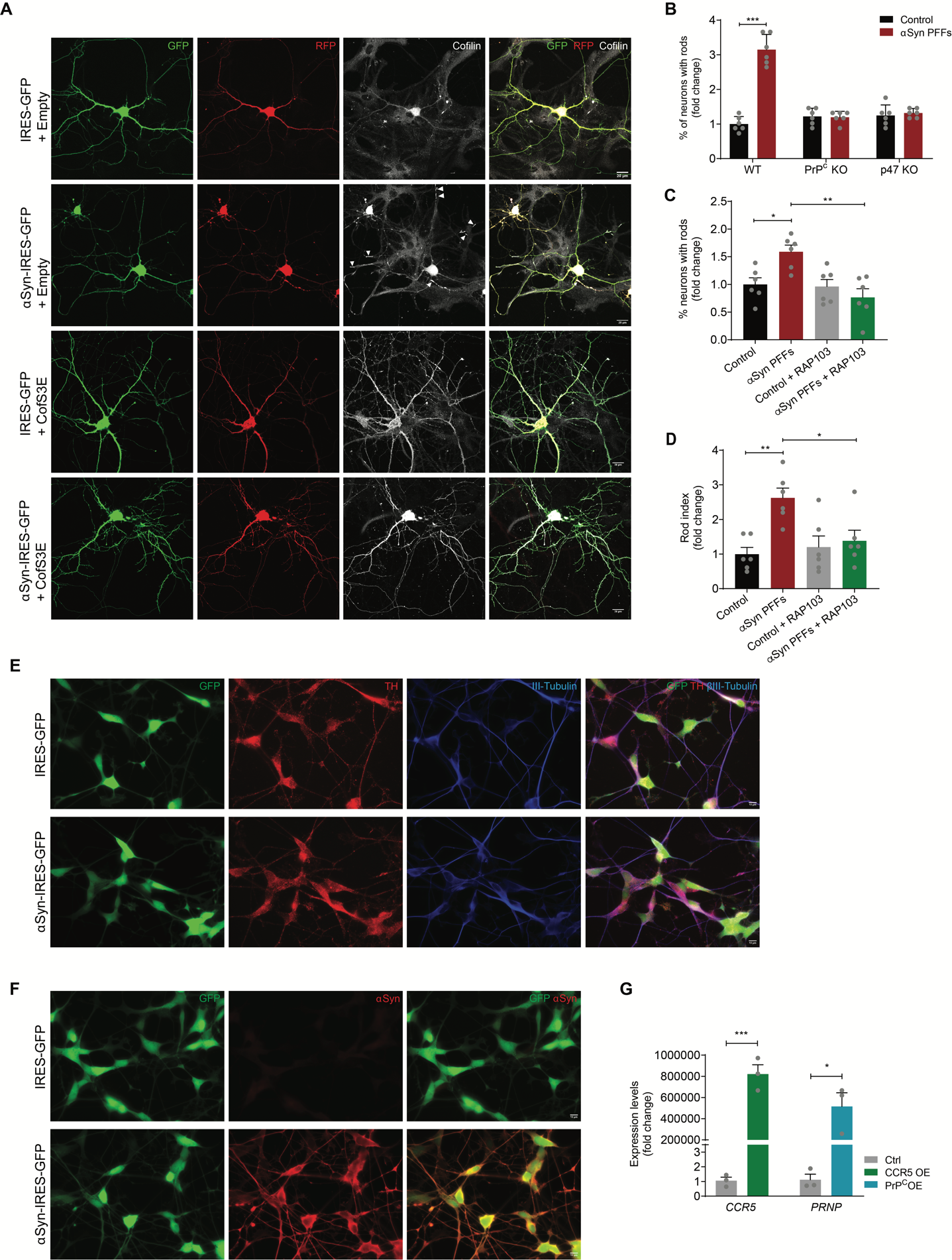
**

**Supplementary Fig. S4 - αSyn-induced rod formation is mediated by PrP^C^, NOX and CCR5.**

**A** Representative images of DIV14 hippocampal neurons overexpressing GFP or αSyn either with pmRFP-N1 or cofilin-S3E and immunostained for cofilin (white). Arrowheads indicate cofilin-actin rod structures. Scale bar: 20μm. **B** Quantification of the percentage of neurons with rods (fold change relative to control) in DIV7 hippocampal neurons from WT, PrPC KO or p47 KO mice pre-treated with control (PBS) or αSyn PFFs 1 μg/mL for 24 h. Data represent mean±SEM (n=6 independent samples/condition with ≥100 neurons/sample). ****p<0.0001 by Two-way ANOVA with Sidak’s multiple comparisons test. **C** Quantification of the percentage of neurons with rods (fold change relative to control) in DIV7 hippocampal neurons pre-treated with control (PBS) or αSyn PFFs 1 μg/ml and with control (H_2_O) or RAP103 (50 pM) for 24 h. Data represent mean±SEM (n=6 independent samples/condition with ≥100 neurons/sample). *p<0.05, **p<0.01 by One-way ANOVA with Tukey’s multiple comparison test. **D** Quantification of the rod index (shown as fold change relative to control) in DIV14 hippocampal neurons pre-treated with control (PBS) or αSyn PFFs 1 μg/ml at DIV7 and with control (H_2_O) or RAP103 (50 pM) for 24 h before fixation. Data represent mean±SEM (n=6 independent samples/condition with ≥100 neurons/sample). *p<0.05, **p<0.01 by One-way ANOVA with Tukey’s multiple comparison test. **E** Representative images of differentiated SH-SY5Y cells expressing GFP or αSyn and immunostained for TH (red) and βIII-tubulin (blue). Scale bar: 10 μm. **F** Representative images of differentiated SH-SY5Y cells expressing GFP or αSyn and immunostained for αSyn (red). Scale bar: 10 μm. **G** qPCR data for *CCR5* and *PRNP* gene expression SH-SY5Y in cells overexpressing CCR5 or PrP^C^. Data shown as fold change in relation to control cells. *ACTB* was used as a reference gene. Data represent mean ± SEM (n=3 independent experiments). *p<0.05, ***p<0.001 by Student’s t test.
