# Supplementary figures and images for: α-Synuclein triggers cofilin pathology and dendritic spine impairment via a PrPC-CCR5 dependent pathway
Source: Cell Death Dis. 2024 Apr 13;15(4):264. doi: 10.1038/s41419-024-06630-9 (PMC11016063; doi:10.1038/s41419-024-06630-9)

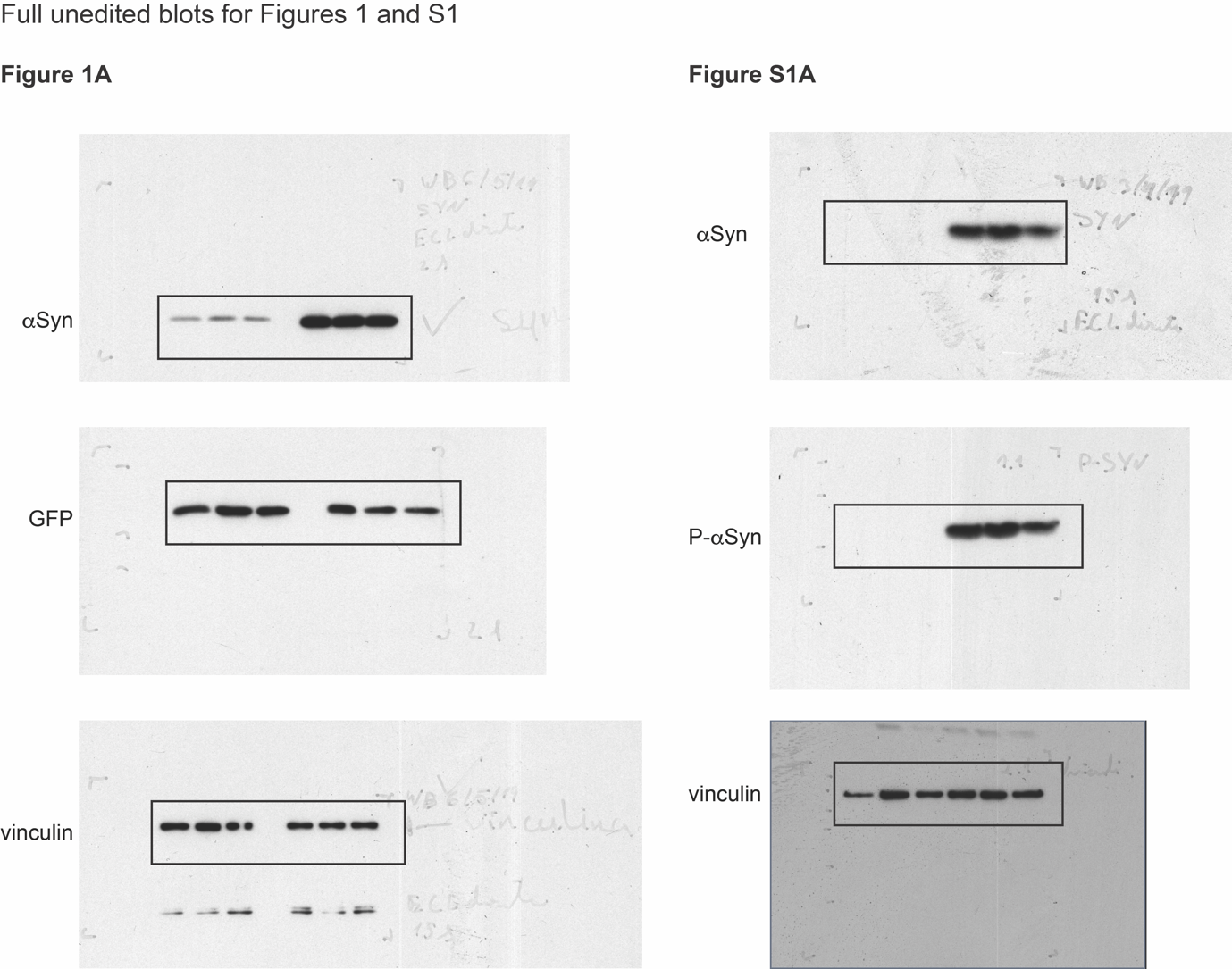


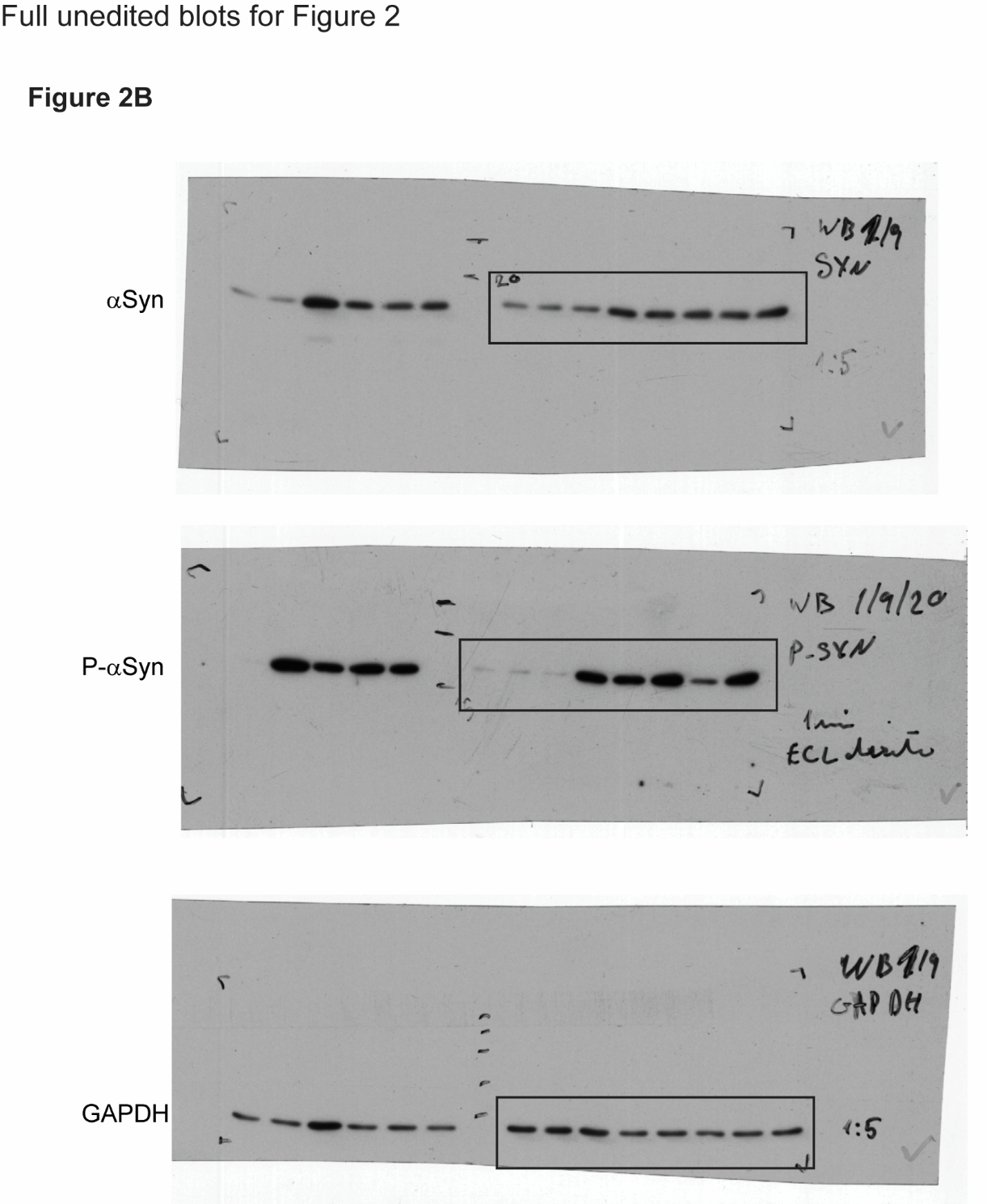

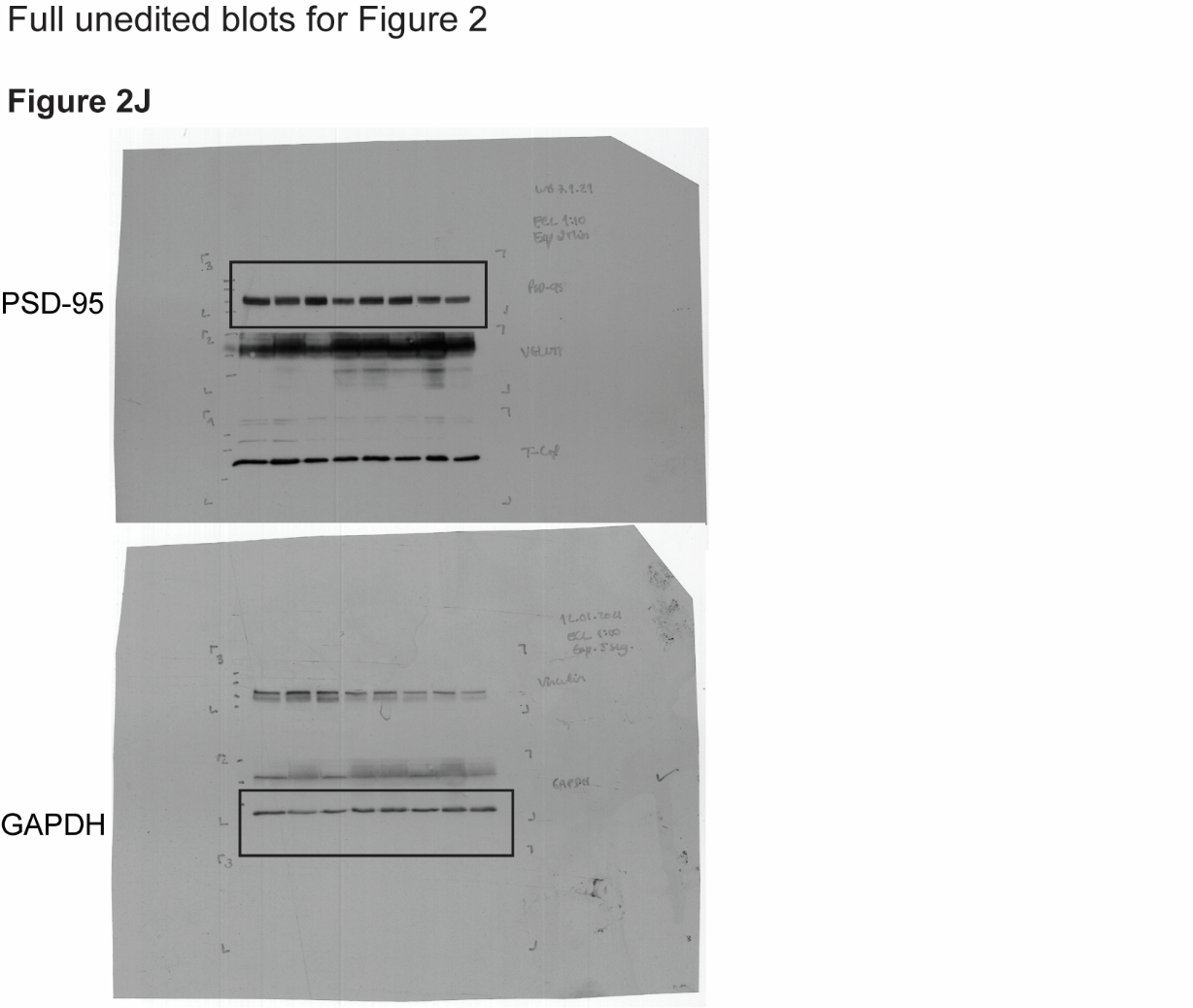

Supplement: Supplementary file 2 — Original Data File [file 41419_2024_6630_MOESM2_ESM.docx]
